# Non Invasive Ventilation Away from ICU

**Published:** 2012-01-18

**Authors:** Alessandro Vatrella, Immacolata Fabozzi

**Affiliations:** Section of Respiratory Disease, Department of Medicine and Surgery, University of Salerno, Italy

We read with great interest the paper by De Robertis and colleagues on non-invasive ventilation (NIV) published on the first issue of this Journal (1). The Authors discussed the rationale for an early NIV approach in non ICU settings and proposed organizational solutions to offer to a large population of patients NIV in a safe way under the supervision of a medical emergency team.

In the last two decades non-invasive mechanical ventilation has become a major therapy of acute respiratory failure. It is a safe, versatile and effective technique that can prevent side effects and complications associated with endotracheal intubation. In fact, compared to invasive ventilation, NIV is associated with a lower risk of nosocomial infections, less antibiotic use, shorter lengths of stay in ICU, and lower mortality (2).

Non-invasive ventilation can be used in a wide range of disorders that lead to acute respiratory failure. The benefit of NIV is very well established in ventilatory failure resulting from acute exacerbations of chronic obstructive pulmonary disease (COPD) as well as in acute cardiogenic pulmonary edema. Moreover, indications for NIV are still increasing, especially in hypoxemic respiratory failure in immunocompromised patients, in ventilator weaning and in the post-operative setting. As a result, growing evidence of the benefits of NIV in multiple indications has led to a progressively more employ in many countries, but frequency of its use is highly variable (3).

The success of NIV relies on several factors, including the type and severity of acute respiratory failure, the underlying disease, the location of treatment, and the experience of the team. The time factor is also of a crucial importance.

For safety reasons, as well as to improve the chances of success, NIV requires an appropriate environment with the presence of staff with training and expertise in NIV, and in adequate numbers for 24/24 cover, facilities for monitoring, and rapid access to endotracheal intubation and invasive ventilation (4).

Obviously, the ICU setting fits all these criteria. Unfortunately, the availability of intensive care beds is often inadequate to the number of patients in acute respiratory failure that need NIV treatment. In order to alleviate this problem the use of NIV in other settings is becoming common and several authors have reported the application of NIV outside the ICU in non-intensive wards.

In a multicenter controlled trial Plant et al. suggested that, with adequate staff training, NIV can be applied with benefit in the general respiratory ward with the usual ward staff (5). Furthermore, North-American surveys have shown that NIV outside the ICU was becoming a widespread practice (6–8). This observation raises the issues of staffing and training in non-intensive wards where nurse to patient ratio is commonly modest.

A pilot observational study by Cabrini et al. provided new interesting data regarding the efficiency and safety of NIV management outside the ICU by a medical emergency team (MET) (9). The role of a MET is to identify and treat hospitalized patients with physiological instability and, therefore, prevents further adverse events. The results of the study by Cabrini et al. are quite encouraging since 77% of patients avoided intubation with few complications and a sustainable work-load for the MET. However, the relevant results of this study, conducted by highly experienced investigators, are the fruit of a very long process and cannot be translated elsewhere without caution.

In conclusion, we agree that initiation and continuation of NIV outside ICUs under the supervision of a MET staffed by anaesthesiologists expert in NIV is an attracting safe and less expensive alternative to invasive ventilation. Nonetheless, we believe that the successful development of non-invasive ventilation in non-intensive wards depends on many variables. The most important is a multidisciplinary approach that incorporate experience and education not limited to the critical care setting.
